# Outcomes of Fluorescence-Guided vs White Light Resection of Glioblastoma in a Single Institution

**DOI:** 10.7759/cureus.42695

**Published:** 2023-07-30

**Authors:** Li Siang Wong, Jerome St. George, Kevin Agyemang, Athanasios Grivas, Deborah Houston, Sin Yee Foo, Thomas Mullan

**Affiliations:** 1 General Medicine, Royal Alexandra Hospital, Paisley, GBR; 2 Neurosurgery, Queen Elizabeth University Hospital, Glasgow, GBR; 3 Radiology, Queen Elizabeth University Hospital, Glasgow, GBR

**Keywords:** fluorescence-guided surgery, neurosurgery, 5-aminolevulinic acid, 5-ala, glioblastoma

## Abstract

Background

Glioblastoma (GBM) is the most common malignant primary brain tumour and confers a very poor prognosis. Maximal safe resection of tumour is the goal of neurosurgical intervention and may be more easily achieved through the use of surgical adjuncts such as fluorescence-guided surgery (FGS). 5-Aminolevulinic acid (5-ALA) accumulates in GBM tissue and fluoresce red, distinguishing tumour cells from the surrounding tissue and therefore making resection easier. 5-ALA-guided resection in GBM has been shown to increase resection rates and prolong progression-free survival without impacting post-operative morbidity. Radiotherapy and concomitant chemotherapy also improve survival in GBM. Other factors such as patient age and molecular status of the tumour also impact prognosis.

Aims

The aim of this study was to compare the outcomes of 5-ALA vs white light-guided resection for glioblastoma in the west of Scotland.

Methods

This was a retrospective analysis of baseline characteristics (age, sex, tumour molecular markers, radiotherapy, chemotherapy, anatomical location of tumour and treatment group) and outcomes (mortality, survival, degree of resection and performance status) of 239 patients who underwent primary resection of glioblastoma over a four-year period (2017-2020). A variety of statistical methods were used to analyse the relationship between each variable and surgical technique; multivariate Cox regression and the Kaplan-Meier method were used in survival analysis.

Results

5-ALA-guided resection substantially improved resection rates (74.0% vs 40.2%). Mortality at 15 months was 5.1% lower in the 5-ALA group (52.0% vs 57.1%, p = 0.53), and patients lived an average of 68 days longer compared to the white light group (444 days vs 376 days, p = 0.21). There were negligible differences between treatment groups in terms of post-operative performance status (PS) and post-operative complications. In our multivariate Cox regression model, six factors were statistically significant at a level of p ≤ 0.05: age, radiotherapy, chemotherapy, O(6)-methylguanine-DNA methyltransferase (MGMT) methylation, anatomical location and >90% resection. Receiving chemotherapy and radiotherapy, MGMT methylation and undergoing >90% resection conferred a survival benefit at 15 months. Older age and multi-focal disease were related to a worsened mortality rate. Undergoing radiotherapy and maximal resection were the two greatest predictors of improved survival, reducing mortality risk by 58% and 51%, respectively.

Conclusion

5-ALA-guided resection improved resection rates without impacting post-operative morbidity. 5-ALA-guided resection was associated with improved survival and lower mortality rate, but this was not statistically significant. Receiving chemoradiotherapy, MGMT methylation and undergoing maximal resection conferred a survival benefit, whilst older age and multi-focal disease were associated with a poorer prognosis.

## Introduction

Glioblastoma (GBM) is the most common malignant primary brain tumour and accounts for approximately 50% of gliomas [1,2]. Despite recent advances, GBM still confers a very poor prognosis, with a mean survival of around 11-15 months with treatment [3,4]. Maximal safe resection of contrast-enhancing tumour (resection of > 90% of tumours) is the goal of neurosurgical intervention [5,6]. However, GBM is aggressive and often invades multiple lobes of the brain, making gross total resection difficult. Resection may be more easily achieved through the use of surgical adjuncts such as fluorescence-guided surgery (FGS). 5-Aminolevulinic acid (5-ALA) is a porphyrin which, when administered orally between two and four hours pre-operatively, accumulates in GBM tissue and causes tumour cells to fluoresce red under blue light microscopy. It distinguishes tumour cells from the surrounding tissue thereby making pathological tissue more easily resectable [7-9]. Stummer et al. first demonstrated the efficacy of 5-ALA-guided resection in GBM: 5-ALA FGS was associated with greater resection rates which conferred a longer time to disease progression and longer overall survival [10]. This has been further demonstrated in various other studies which have shown that 5-ALA FGS improves resection rates, which subsequently improves survival and performance status at six months [11-13].

Radiotherapy and concomitant chemotherapy improve survival in patients with GBM [14,15]. Post-operatively, only patients with a Karnofsky performance status (PS) ≥ 70 are offered radiotherapy with concomitant temozolomide [16]. Molecular markers also play an important role in determining prognosis in GBM. Patients with mutations in isocitrate dehydrogenase (IDH) 1 and 2 enzymes have a favourable prognosis [17-19]. O(6)-methylguanine-DNA methyltransferase (MGMT) gene methylation is also associated with longer progression-free and overall survival in patients with GBM; this survival benefit is further enhanced by the addition of temozolomide chemotherapy to radiotherapy as MGMT gene methylation makes tumour cells more susceptible to chemotherapy [20-22]. In this study, we compare outcomes of 5-ALA- vs white light-guided resection for glioblastoma in the west of Scotland. This paper was previously presented as an abstract at the Society of British Neurosurgeons Conference on September 22, 2021.

## Materials and methods

This is a single-centre, retrospective cohort study of baseline characteristics and outcomes of 239 patients who underwent primary resection of newly diagnosed GBM over a four-year period (2017-2020) in a single neurosurgical centre in the west of Scotland. The patient database was provided by two researchers (AG and DH). All aspects of data collation and analysis were conducted by two researchers (LSW and TM), with additional input from a senior researcher (KA) who also cross-checked 10% of the data. Any discrepancies were resolved through consensus, with the senior researcher (ESG) making final decisions.

Patient selection

**Figure 1:**
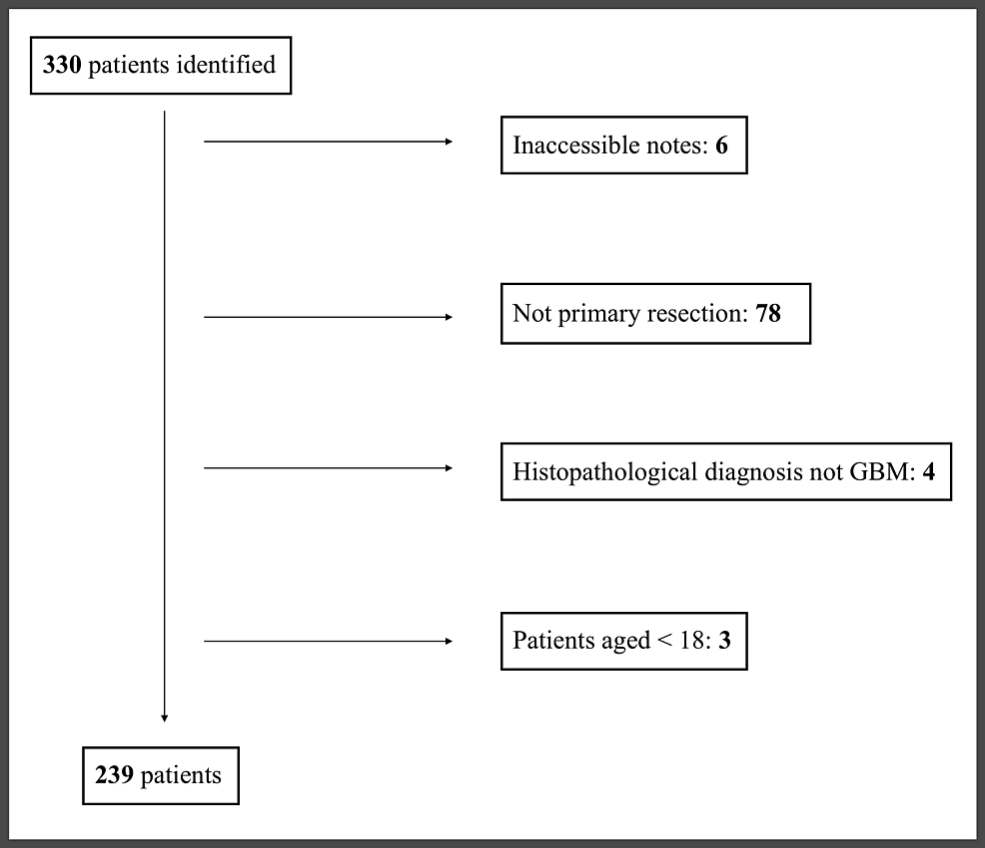
Inclusion and exclusion of patients undergoing GBM resection. GBM: glioblastoma.

Patients were identified from a single database comprising patients who were presented at the neuro-oncology multi-disciplinary team (MDT) meeting at the Queen Elizabeth University Hospital. A total of 330 patients were identified across the four-year period (Figure 1). All patients aged ≥ 18, undergoing primary resection of GBM (histopathological diagnosis of WHO Grade IV astrocytoma) with accessible medical records, were included in this study. 

Data collection

Demographic and clinical data were collected on each patient using medical records accessed via Clinical Portal and TrakCare. Fifteen factors were analysed for each patient (Table 1). Patients’ Community Health Index (CHI) numbers were securely stored on a National Health Service (NHS) computer. Select variables are described below. Where resection rates were unreported or unavailable in patient notes (n = 29), a senior radiologist (SF) reviewed pre- and post-operative T1-weighted MRI scans and measured the degree of resection using volumetric analysis on Picture Archiving Communications System (PACS) software (Philips, Amsterdam, Netherlands) (n = 23). Six patients did not have imaging available. Intra-operative imaging techniques were not used in either treatment group. Performance status was analysed pre- and post-operatively. This allowed change in performance status (increase or no change/decrease) to be assessed. Performance status was also dichotomised into high (≥ 2) and low (≤ 1) to allow for statistical analysis as a categorical variable. As the data were analysed in 2021, mortality was censored at 15 months to allow sufficient follow-up time for patients included in the 2020 analysis, with the mean survival of GBM being 11-15 months [3,4]. Sixteen patients were not included in the survival analysis due to missing data.

**Table 1:** Data collected on each patient and their definitions. PS: performance status, 5-ALA: 5-aminolevulinic acid, MGMT: O(6)-methylguanine-DNA methyltransferase, IDH: isocitrate dehydrogenase.

Variable	Categories	Definition
Age	Continuous variable	Age at the time of surgery
Sex	Male	Male or female at birth
	Female	
Radiotherapy	Received radiotherapy	Patient received standard radiotherapy treatment, as recorded in the clinical oncology notes.
	Did not receive radiotherapy	
Chemotherapy	Received temozolomide	Patient received standard chemotherapy treatment with temozolomide, as recorded in the clinical oncology notes.
	Did not receive temozolomide	
MGMT methylation	MGMT methylated	Tumour cells containing methylated MGMT, as reported in the pathology notes.
	MGMT not methylated	
IDH 1/2 mutation	IDH 1/2 mutated	Tumour cells containing IDH 1/2 mutations, as reported in the pathology notes.
	IDH 1/2 not mutated	
Anatomical location of tumour	Single lobe (frontal lobe, parietal lobe, occipital lobe, temporal lobe, cerebellum)	The location of the tumour as reported in the patient’s notes. Tumours were classified as ‘multiple lobes’ if the tumour extended into more than one lobe, as reported in the radiology notes.
	Multiple lobes	
Treatment group	5-ALA	Resection was carried out under white light resection or using 5-ALA fluorescence-guided resection, as reported in the neurosurgery operation notes.
	White light	
WHO performance status	Pre-operative WHO performance status	WHO performance status pre- and post-operatively, as reported in the patient’s notes. PS was also categorised into high (≥ 2) and low (≤ 1).
	Post-operative WHO performance status	
Change in WHO performance status	Increase	The difference in WHO performance status between post-operative and pre-operative assessment, as recorded in the patient's notes.
	No change or decrease	
Pre-operative neurological deficit	No deficit	Neurological deficit recorded pre-operatively in the patient's notes.
	Pre-operative deficit	
New post-operative neurological deficit	No new post-operative deficit	Neurological deficit recorded post-operatively (in addition to pre-operative deficit) in the patient's notes.
	New post-operative deficit	
Degree of resection	> 90% resection	Degree of resection of contrast-enhancing tumour on T1 MRI, measured using volumetric analysis on Picture Archiving Communications System (PACS) software, as reported by a radiologist in the patient’s notes.
	< 90% resection	
Mortality (15 months)	Alive	Mortality at 15 months, calculated from the date of surgical resection.
	Deceased	
Survival (days)	Continuous variable	Time to death, calculated in days, from the date of surgical resection. Censored at 15 months.

Statistical analyses 

Descriptive statistics were analysed for each variable in our dataset to provide a summary of the data’s variability and distribution. For our univariable analysis, we used different statistical methods depending on the nature of the variables. Chi-Square test was used to analyse the association between categorical variables and mortality. To assess the association between categorical variables and survival, as well as between continuous variables and mortality, we employed analysis of variance (ANOVA). Furthermore, we utilised Pearson correlation coefficients to assess the correlation between continuous variables and survival. These analyses provided insights into the potential factors that may influence mortality and survival in our study population, and they served as a foundation for our subsequent multivariable analysis using Cox regression and Kaplan-Meier methods.

We employed a multivariate Cox regression model to evaluate the association between mortality and 12 covariates, including age, sex, radiotherapy, chemotherapy, MGMT methylation, IDH mutation, anatomical location, treatment group, pre-operative PS, Change in PS, new post-operative neurological deficit and degree of resection. All assumptions of the model were met. We present the hazard ratios (HR) and their corresponding standard errors. The statistical significance of the HRs was assessed using a significance threshold value of p ≤ 0.05. Any covariate with a p-value below this threshold was considered to have a significant association with mortality. 

In addition to Cox regression analysis, the Kaplan-Meier method was used to perform survival analysis with data censored at 15 months. We constructed a Kaplan-Meier curve that demonstrates the estimated survival probabilities over time for each treatment group. The log-rank test was utilised to detect statistically significant differences between the treatment groups. All analyses were conducted using Minitab 19 (Pennsylvania State University, State College, USA) and SPSS (Version 26, IBM, New York, USA).

## Results

There were 239 patients who underwent primary resection of GBM between 2017 and 2020 who fulfilled the inclusion criteria: 50 (21%) were conducted using 5-ALA FGS and 189 (79%) were under white light.

Baseline characteristics 

**Figure 2:**
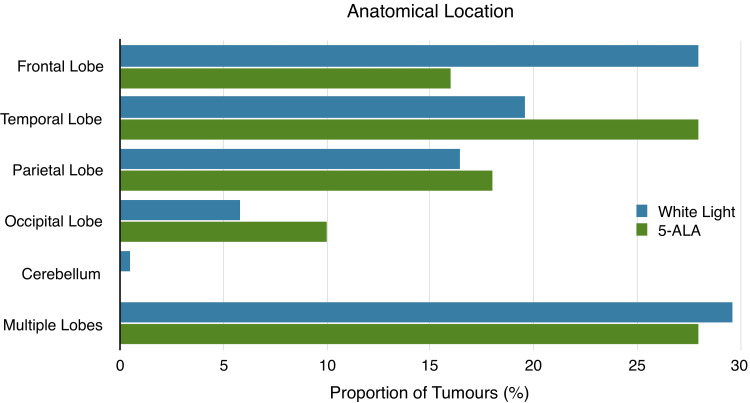
Anatomical location of tumours in the white light and 5-ALA groups. 5-ALA: 5-aminolevulinic acid.

Patients in the 5-ALA group were slightly younger, and a greater proportion were male. Both groups received similar post-operative treatment and had similar proportions of pre-operative deficits and molecular mutations. Tumour location was also similar in each treatment group (Figure 2).

A larger proportion of patients in the 5-ALA group had a low pre-operative PS (84.0% vs 66.7%; Table 2).

**Table 2:** Baseline characteristics, tumour molecular markers and post-operative management of patients undergoing primary glioblastoma (GBM) resection. Missing data: ^a^three patients with missing data, ^b^one patient with missing data, ^c^one patient with missing data, ^d^two patients with missing data, and ^e^two patients with missing data. PS: performance status, 5-ALA: 5-aminolevulinic acid, MGMT: O(6)-methylguanine-DNA methyltransferase, IDH: isocitrate dehydrogenase.

Patient characteristics	White light (n = 189)	5-ALA (n = 50)
Age (median)	60	58
Sex	Female: 88 (46.6%)	Female: 17 (34.0%)
	Male: 101 (53.4%)	Male: 33 (66.0%)
Pre-operative PS	PS ≤ 1: 126 (66.7%)^a^	PS ≤ 1: 42 (84.0%)
	PS ≥ 2: 60 (31.7%)^a^	PS ≥ 2: 8 (16.0%)
Pre-operative deficit	174 (92.0%)	44 (88.0%)
Tumour molecular markers	White light (n = 189)	5-ALA (n = 50)
MGMT methylated	86 (45.5%)	18 (36.0%)^b^
IDH 1/2 mutation	17 (9.0%)	2 (4.0%)^c^
Post-operative treatment	White light (n = 189)	5-ALA (n = 50)
Radiotherapy	139 (73.5%)^d^	40 (80.0%)
Chemotherapy	134 (70.9%)^e^	41 (82.0%)

Outcomes

In our univariable analysis, 10 variables were independently associated with prolonged survival (age, radiotherapy, chemotherapy, MGMT methylation, treatment group, anatomical location, pre-operative PS, post-operative PS, change in PS and > 90% resection). Five variables conferred a survival benefit at 15 months (age, radiotherapy, chemotherapy, MGMT methylation and > 90% resection; Tables 3, 4).

**Table 3:** Univariable association between analysed data points and survival outcome measures. p values have been rounded to two decimal places. PS: performance status, ANOVA: analysis of variance, MGMT: O(6)-methylguanine-DNA methyltransferase, IDH: isocitrate dehydrogenase.

Variable	Survival (days)		Mortality (15 months)	
	ANOVA (F)	p value	Chi Sq (χ^2^)	p value
Sex	0.70	0.79	0.42	0.75
Radiotherapy	66.91	< 0.01	123.01	< 0.01
Chemotherapy	42.37	< 0.01	119.91	< 0.01
Treatment group	3.88	0.05	1.69	0.51
MGMT methylation	3.98	0.05	6.38	< 0.01
IDH mutation	2.48	0.12	15.37	0.06
Anatomical location	9.98	< 0.01	15.29	0.06
Pre-operative PS	4.45	0.04	12.45	0.09
Post-operative PS	12.47	< 0.01	13.94	0.07
Change in PS	4.33	0.04	9.79	0.13
New deficit	1.99	0.16	0.45	0.74
> 90% resection	15.80	< 0.01	0.01	0.01

**Table 4:** Univariable association between age and survival outcome measures. p values have been rounded to two decimal places. ANOVA: analysis of variance.

Variable	Survival (days)		Mortality (15 months)	
	Pearson Correlation Coefficient (r)	p value	ANOVA (F)	p value
Age	-0.21	< 0.01	14.47	< 0.01

Prolonged survival was seen in younger patients, those with single lobe disease, a low pre- and post-operative PS and those with a decrease in PS post-operatively. This was also the case for patients who received chemotherapy and radiotherapy, had MGMT mutations, underwent 5-ALA-guided resection and achieved > 90% resection. Similar correlations were observed between age, radiotherapy, chemotherapy, MGMT methylation, > 90% resection and mortality. Age had a negative correlation with survival but a positive correlation with mortality. Patients who received chemotherapy and radiotherapy had MGMT mutations and who underwent > 90% resection had lower mortality.

**Figure 3:**
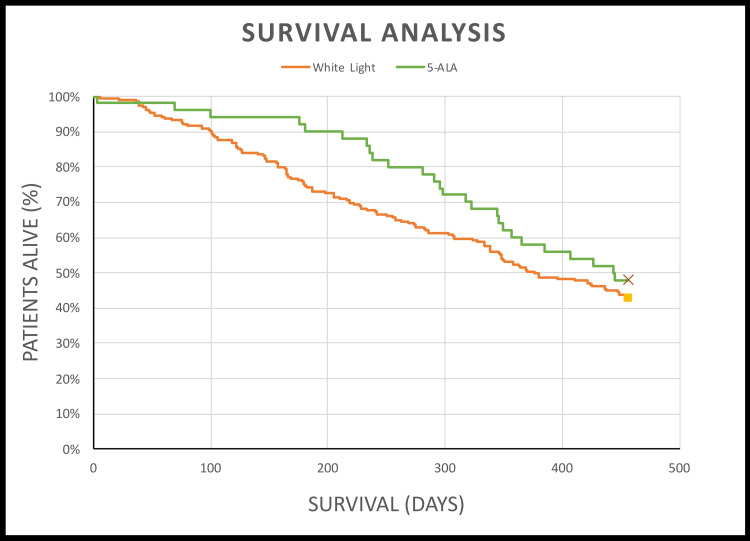
Kaplan-Meier curve (white light vs 5-ALA). Log-rank test: p = 0.28. p values have been rounded to two decimal places. 5-ALA: 5-aminolevulinic acid.

5-ALA FGS improved resection rates, with 33.8% more patients undergoing complete resection (> 90% resection) in the 5-ALA group (74.0% vs 40.2%). There were negligible differences between treatment groups in terms of post-operative PS and post-operative complications. Mortality at 15 months was 5.1% lower in the 5-ALA group (52.0% vs 57.1%, Chi-Square test, p = 0.53), and patients lived an average of 68 days longer compared to the white light group (444 days vs 376 days, log-rank test, p = 0.21; Figure 3 and Table 5).

**Table 5:** Post-operative morbidity, resection rates and survival outcomes in the white light and 5-ALA groups. Missing data: ^a^five patients with missing data, ^b^two patients with missing data, ^c^one patient with missing data, ^d^five patients with missing data, and ^e^one patient with missing data. PS: performance status, 5-ALA: 5-aminolevulinic acid.

WHO performance status	White Light (n = 189)	5-ALA (n = 50)
Post-operative PS	PS ≤ 1: 116 (61.4%)^a^	PS ≤ 1: 35 (70%)
	PS ≥ 2: 68 (36.0%)^a^	PS ≥ 2: 15 (30%)
Increase in PS	50 (26.4%)^a^	16 (32.0%)
Neurological deficit	White light (n = 189)	5-ALA (n = 50)
New post-operative deficit	32 (16.9%)^b^	11 (22.0%)^c^
Degree of resection	White light (n = 189)	5-ALA (n = 50)
> 90% resection	76 (40.2%)^d^	37 (74.0%)^e^
Survival outcomes	White light (n = 189)	5-ALA (n = 50)
Mortality (15 months)	57.1%	52.0%
Median survival (days)	376	444

Six factors were statistically significant at a level of p ≤ 0.05 in our multivariate Cox regression model: age, radiotherapy, chemotherapy, MGMT methylation, anatomical location and > 90% resection (Table 6).

**Table 6:** Multivariate Cox regression model with associated hazard ratios. p values have been rounded to two decimal places. PS: performance status, MGMT: O(6)-methylguanine-DNA methyltransferase, IDH: isocitrate dehydrogenase.

Variable	Mortality (15 months)	
	Hazard ratio (HR) and 95% confidence intervals (CIs)	p value
Age	1.02 (1.01, 1.04)	0.01
Sex	0.73 (0.50, 1.07)	0.10
Radiotherapy	0.42 (0.27, 0.66)	< 0.01
Chemotherapy	0.60 (0.38, 0.92)	0.02
Treatment group	0.88 (0.55, 1.39)	0.58
MGMT methylation	0.52 (0.35, 0.78)	< 0.01
IDH mutation	0.90 (0.37, 2.7)	0.81
Anatomical location	2.09 (1.39, 3.13)	< 0.01
Pre-operative PS	1.00 (0.67, 1.51)	0.98
Change in PS	1.16 (0.78, 1.73)	0.47
New deficit	1.02 (0.61, 1.69)	0.94
> 90% resection	0.49 (0.33, 0.71)	< 0.01

Receiving chemotherapy and radiotherapy, MGMT methylation and undergoing > 90% resection conferred a survival benefit at 15 months. Older age and multi-lobe disease were related to a worsened mortality rate. Undergoing radiotherapy and maximal resection were the two greatest predictors of improved survival, reducing mortality risk by 58% and 51%, respectively.

## Discussion

The National Institute for Health and Care Excellence (NICE) advise utilising 5-ALA FGS in patients who have radiologically enhancing suspected high-grade glioma and when resection of all enhancing tumour is possible [16]. The decision may also be influenced by a number of factors including patients’ age and pre-operative PS. In our neurosurgical centre over a four-year period, only 50 (21%) patients underwent 5-ALA-guided resection. These patients were slightly younger, predominantly male and had lower pre-operative PS. There was no clear association between the anatomical location of the tumour and surgical technique (5-ALA vs white light) as there were a similar proportion of patients suffering multi-focal disease in each treatment group (29.6% vs 28.0%). This was a surprising finding given that NICE suggest using 5-ALA if resection of all enhancing tumour is possible (a factor which is very likely to be influenced by a tumour’s anatomical location), and complete resection would be more difficult to achieve in patients with multi-focal disease. The decision to use 5-ALA remains a difficult one and often involves multi-disciplinary discussion involving multiple specialties at neuro-oncology meetings. Different neurosurgical centres use 5-ALA differently, and each centre has its own standard of practice. In our centre, the selection of patients for 5-ALA-guided resection was not consistent. This audit has highlighted the need for standardisation of our practice in terms of the selection of patients. More research and more stringent guidelines are needed in order to identify the subgroup of patients most likely to benefit from 5-ALA-guided resection.

The patient selection remains important as 5-ALA should be used in patients most likely to benefit from fluorescence-guided resection. It is also important to consider the cost-effectiveness of 5-ALA as one 50ml (30mg/ml) vial, the standard dose in our centre, costs approximately £950 [23]. In other European centres, the cost of 5-ALA ranges between €1000 and 2000 per patient, depending on the patients’ weight [24]. From an economic standpoint alone, it would be costly to use 5-ALA in every patient undergoing GBM resection, further highlighting the need for more stringent patient selection.

A survival benefit was observed in the 5-ALA group, with mortality 5.1% lower (Chi-Square test, p = 0.53) and patients living an average of 68 days longer compared to the white light group (log-rank test, p = 0.28). This was reflected in the Kaplan-Meier curve, where the greatest difference was observed between 200 and 300 days post-operatively. Although not statistically significant in this study, a prolonged survival of 68 days may be clinically significant. Treatment group (5-ALA or white light) also did not significantly contribute to our multivariate Cox regression model (HR = 0.88, 95% CI: 0.55-1.39, p = 0.58). In other words, in our model, mortality was not significantly influenced by the type of surgical technique used. It was unclear why, despite greater resection rates, the use of 5-ALA did not significantly impact mortality or survival and, as stated previously, is likely due to the relatively low number of patients in the 5-ALA group.

In our model, receiving chemotherapy (HR = 0.60, 95% CI: 0.38-0.92, p = 0.02), radiotherapy (HR = 0.42, 95% CI: 0.27-0.66, p < 0.01), MGMT methylation (HR = 0.52, 95% CI: 0.35-0.78, p < 0.01) and undergoing maximal resection (HR = 0.49, 95% CI: 0.33-0.71, p < 0.01) reduced mortality by 40%, 58%, 48% and 51%, respectively, consistent with previous research [10-15, 20-22]. Despite previous research also suggesting that mutations in IDH enzymes confer a favourable prognosis [17-19], this was not demonstrable in our study. Older age (HR = 1.02, 95% CI: 1.01-1.04, p = 0.01) and multi-lobe disease (HR = 2.09, 95% CI: 1.39-3.13, p < 0.01) were associated with greater mortality, increasing mortality by 2% and 109%, respectively.

Following a recent audit in 2021, Health Improvement Scotland has set a target for all neurosurgical centres in Scotland to achieve complete resection in at least 40% of patients undergoing GBM surgery [25]. This target will be more easily achieved through the use of fluorescence-guided resection, as greater resection rates were obtained in a significantly larger proportion of patients in the 5-ALA group. However, maximal resection might not have been the intention in all of the patients in the white light group, or indeed the 5-ALA group, as patients with multi-focal disease also underwent 5-ALA-guided resection. Despite this, complete resection rates were similar between our study’s white light group (40%) and Stummer’s (36%) [10]. Following this audit, the intent of surgical resection should be documented at the neuro-oncology MDT as it was not explicitly stated in patients’ notes.

Greater resection rates may come at the cost of worsened post-operative morbidity [11]. Stummer also reported transient neurological deficits following 5-ALA FGS up to one week post-operatively that improved after six weeks. This effect was observed in a similar proportion of patients in each treatment group (43% vs 43%, p = 0.3) [10]. In the present study, greater resection rates in the 5-ALA group were not associated with a significant increase in post-operative morbidity. There were negligible differences in PS and neurological deficits between treatment groups, highlighting the safety of 5-ALA-guided resection in a west of Scotland population. The risk of worsened morbidity might be mitigated through the use of intra-operative monitoring such as awake craniotomy or imaging modalities, such as intra-operative magnetic resonance imaging (iMRI), both of which may be used instead of or alongside 5-ALA FGS [26,27]. However, such adjuncts are expensive, labour-intensive and time-consuming and would have been especially difficult to implement during the COVID-19 pandemic.

Importantly, the proportion of patients in each group that received concomitant chemotherapy and radiotherapy (which are only offered to patients with post-operative PS > 70) were similar. This is a significant finding as the aim of treatment in GBM is to preserve the quality of life and prolong survival, which can be best achieved with a low post-operative PS and access to chemo- and radiotherapy. 

In addition to the risk of increased post-operative morbidity, common adverse effects of 5-ALA (termed substance-specific side effects) include photosensitivity reaction, hypotension and nausea. Side effects secondary to the combination of surgery, anaesthesia and 5-ALA (termed procedure-related side effects) include anaemia, thrombocytopenia, leucocytosis and transient liver disorders [28]. The administration of 5-ALA and surgical resection using fluorescence also adds time to the surgical procedure.

Following the World Health Organisation’s (WHO) publication of the classification of tumours of the central nervous system (CNS) in 2021 [29], the term glioblastoma multiforme, synonymous with grade IV astrocytoma, is no longer used. Instead, tumours are classified based on molecular genetics, such as IDH mutation status, and histopathological grade. Previously, all grade IV astrocytomas were referred to as glioblastoma. However, the term glioblastoma is now reserved for grade IV astrocytomas, IDH-wildtype tumours. Tumours which are grade IV astrocytomas, IDH-mutant, which were previously also classed as glioblastomas, are now regarded as a separate entity. In our study, due to data collection taking place prior to the publication of the WHO 2021 classification of CNS tumours, glioblastomas are defined as grade IV astrocytomas.

Our study had various strengths: our internal validation steps ensured consistent data collation and analysing data from a single centre allowed direct conclusions to be drawn. To our knowledge, this is the first study to validate the efficacy and safety of 5-ALA-guided resection in a west of Scotland population. Some limitations to the data extraction of this audit included the retrospective design of the study with limited numbers in the 5-ALA cohort, making statistical differences between the groups more difficult to detect. There were various data missing from patients' notes, particularly the radiology reports which were used to analyse the degree of resection. The effect of this was minimised by having a senior radiologist review patients' scans if the radiology reports were unavailable. In this study, PS was reported as WHO PS grades and not Karnofsky PS scores, which are used in the NICE guidelines. However, a WHO PS < 2 equates to Karnofsky PS score of ≥ 70 [30]. We also analysed the change in PS rather than the absolute values themselves, thereby mitigating the issue. It was also unclear whether the neurological deficits that were observed in either treatment group were transient or permanent as we analysed post-operative PS and neurological deficit one week post-operatively.

## Conclusions

The use of 5-ALA-guided resection in a west of Scotland population significantly improved resection rates without impacting post-operative morbidity. Receiving chemoradiotherapy, MGMT methylation and undergoing maximal resection conferred a survival benefit, whilst older age and multi-lobe disease were associated with a poorer prognosis. Undergoing radiotherapy and maximal resection were the two greatest predictors of improved survival, reducing mortality risk by 58% and 51%, respectively.

Treatment group (5-ALA vs white light resection) was not significantly associated with improved mortality or prolonged survival in our Cox regression models. Lower mortality and an improved survival rate were observed in the 5-ALA group, with mortality 5.1% lower and patients living an average of 68 days longer, but this was not statistically significant at a level of p ≤ 0.05.
